# Dexamethasone Attenuates Oncostatin M Production via Suppressing of PI3K/Akt/NF-κB Signaling in Neutrophil-like Differentiated HL-60 Cells

**DOI:** 10.3390/molecules27010129

**Published:** 2021-12-27

**Authors:** Na-Ra Han, Seong-Gyu Ko, Hi-Joon Park, Phil-Dong Moon

**Affiliations:** 1 College of Korean Medicine, Kyung Hee University, Seoul 02447, Korea; nrhan@khu.ac.kr; 2 Korean Medicine-Based Drug Repositioning Cancer Research Center, College of Korean Medicine, Kyung Hee University, Seoul 02447, Korea; epiko@khu.ac.kr; 3 Department of Preventive Medicine, College of Korean Medicine, Kyung Hee University, Seoul 02447, Korea; 4 Department of Anatomy & Information Sciences, College of Korean Medicine, Kyung Hee University, Seoul 02447, Korea; acufind@khu.ac.kr; 5 Center for Converging Humanities, Kyung Hee University, Seoul 02447, Korea

**Keywords:** oncostatin M, dexamethasone, neutrophil-like differentiated HL-60 cells, PI3K, Akt, NF-κB

## Abstract

Oncostatin M (OSM) plays a role in various inflammatory reactions, and neutrophils are the main source of OSM in pulmonary diseases. However, there is no evidence showing the mechanism of OSM production in neutrophils. While dexamethasone (Dex) has been known to exert anti-inflammatory activity in various fields, the precise mechanisms of OSM downregulation by Dex in neutrophils remain to be determined. Here, we examined how OSM is produced in neutrophil-like differentiated HL-60 cells. Enzyme-linked immunosorbent assay, real-time polymerase chain reaction, and Western blot analysis were utilized to assess the potential of Dex. Granulocyte-macrophage colony-stimulating factor (GM-CSF) stimulation resulted in OSM elevation in neutrophil-like dHL-60 cells. OSM elevation induced by GM-CSF is regulated by phosphatidylinositol 3-kinase (PI3K)/Akt/nuclear factor (NF)-kB signal cascades. GM-CSF stimulation upregulated phosphorylated levels of PI3K or Akt or NF-κB in neutrophil-like dHL-60 cells. Treatment with Dex decreased OSM levels as well as the phosphorylated levels of PI3K or Akt or NF-κB in neutrophil-like dHL-60 cells. Our findings show the potential of Dex in the treatment of inflammatory diseases via blocking of OSM.

## 1. Introduction

Oncostatin M (OSM) was found in supernatants of phorbol 12-myristate 13-acetate (PMA)-treated U-937 monocyte cell line for the first time in 1986 and decreased the growth of A375 melanoma cells and other tumor cells, but not normal fibroblasts [1]. Cytokine OSM belongs to the interleukin (IL)-6 family and is produced in various cell types, such as T cells, macrophages, monocytes, dendritic cells, and neutrophils [2,3,4]. OSM is implicated in a wide range of physiologic and pathologic processes, including remodeling of the extracellular matrix, hematopoiesis, growth modulation of tumor and non-tumor cells, liver regeneration, cardiac remodeling, and inflammatory reactions [5,6]. It also plays a role in various inflammatory reactions. Cytokine OSM is involved in inflammation in rheumatoid arthritis and liver inflammation in hepatocarcinogenesis [7,8]. OSM is also implicated in inflammatory pulmonary diseases, such as allergic rhinitis and asthma [9,10]. West et al. [2] suggested that recombinant OSM treatment promotes inflammatory activity in the human intestinal stroma. OSM can stimulate keratinocytes to produce inflammatory factors and induce skin inflammation with features similar to psoriasis [4]. Recently, Liu and colleagues [11] reported that OSM enhances human keratinocyte-mediated inflammation. Intranasal administration of recombinant OSM induced an inflammatory infiltrate and elevations of inflammatory cytokines and chemokines in mice [12]. Subcutaneous injection of recombinant OSM led to skin inflammation in a murine model [13]. Botelho and colleagues [14] reported that overexpression of OSM resulted in lung inflammation in mice. Pothoven et al. [10] reported that neutrophils are the main source of OSM in inflammatory diseases. However, there is no evidence showing the mechanism of OSM production in neutrophils. We thus investigated how OSM is produced in neutrophil-like differentiated (d) HL-60 cells.

Glucocorticoids are steroid hormones that have a high affinity with intracellular glucocorticoid receptors. The steroid hormone glucocorticoid receptor complex moves into the nucleus where it hinders nuclear factor (NF)-κB driven gene expression [15]. Dexamethasone (Dex) is a synthetic glucocorticoid that exerts very high potency to induce anti-inflammatory effects relative to the endogenously produced cortisol [16]. Numerous studies have suggested that the glucocorticoid Dex has anti-inflammatory activity [17,18,19,20]. It is hence proposed that Dex is an excellent remedy to treat inflammatory disorders, such as acute otitis externa, allergic rhinitis, chronic rhinosinusitis, and asthma [21,22,23,24]. It is already known that dexamethasone is an anti-inflammatory drug suppressing the migration of neutrophils and decreasing lymphocyte colony proliferation, and it already has a wide variety of uses in human medicine [25]. Although the anti-inflammatory potential of Dex is well known, the precise mechanism of OSM down-regulation by Dex in neutrophils has not been fully elucidated.

In the present study, we investigated the mechanism of OSM production as well as the inhibitory effects of Dex on OSM levels in neutrophil-like dHL-60 cells.

## 2. Results

### 2.1. OSM Increases by Granulocyte-Macrophage Colony-Stimulating Factor (GM-CSF) Stimulation in Neutrophil-like dHL-60 Cells

**Figure 1:**
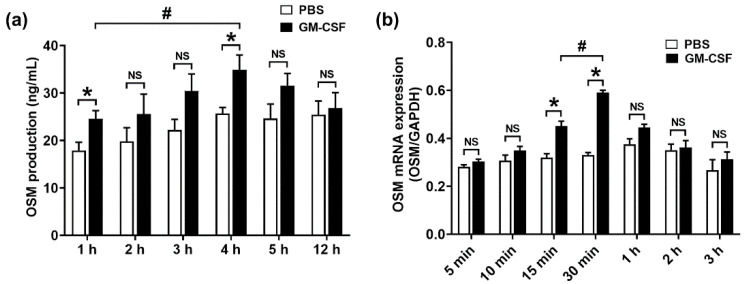
Production and mRNA expression of OSM in neutrophil-like dHL-60 cells. (**a**) dHL-60 cells (5 × 10^5^) were stimulated with GM-CSF (5 ng/mL). OSM levels were measured with the ELISA method. (**b**) dHL-60 cells (1 × 10^6^) were stimulated with GM-CSF (5 ng/mL). OSM mRNA levels were measured with the real-time PCR method. PBS—PBS-treated and unstimulated cells; GM-CSF—PBS-treated and GM-CSF-stimulated cells. Data are presented as the mean ± S.E.M. of three independent experiments. * *p* < 0.05 vs. the PBS-treated and unstimulated cells. ^#^ *p* < 0.05 vs. the indicated group. NS, not significant.

Cross and colleagues [26] revealed that OSM expression increased by GM-CSF stimulation in healthy control blood neutrophils. Furthermore, an increase in OSM protein production resulted from the addition of human GM-CSF in isolated peripheral neutrophils from human subjects [27]. Thus, we examined whether GM-CSF treatment increases OSM production in neutrophil-like dHL-60 cells. When we stimulated neutrophil-like dHL-60 cells with GM-CSF, production levels of OSM were elevated (Figure 1a). Furthermore, we investigated when the production levels of OSM reach their peak in neutrophil-like dHL-60 cells. The OSM levels reached the maximum production 4 h after GM-CSF treatment (Figure 1a). We next examined whether GM-CSF treatment increases OSM mRNA expression in neutrophil-like dHL-60 cells. The OSM mRNA levels reached the maximum expression 1 h after GM-CSF treatment (Figure 1b).

### 2.2. OSM Elevation Induced by GM-CSF Is Mediated by Phosphatidylinositol 3-Kinase (PI3K)/Akt/Nuclear Factor (NF)-kB in Neutrophil-like dHL-60 Cells

**Figure 2:**
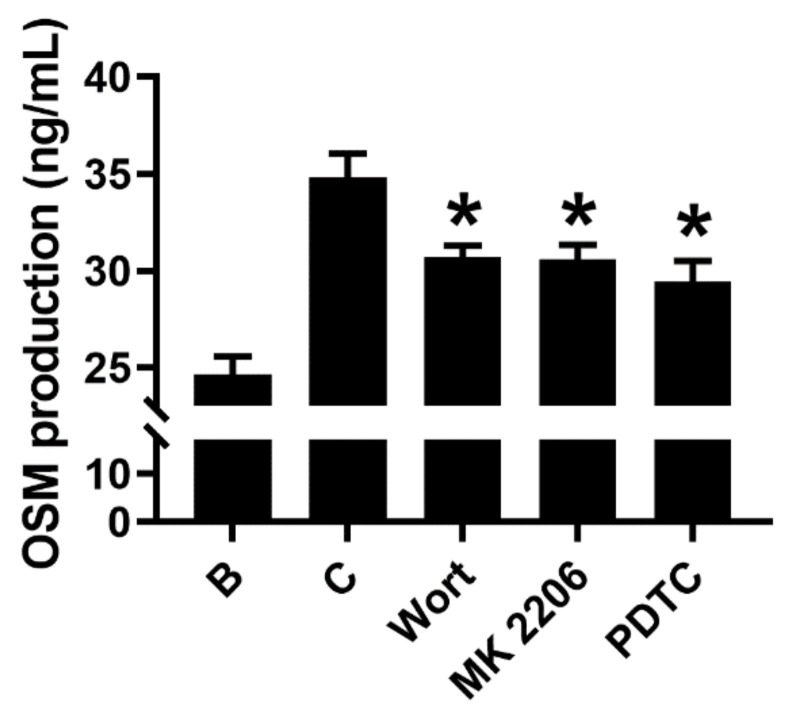
Inhibition of OSM levels by each inhibitor. dHL-60 cells (5 × 10^5^) were pretreated with each inhibitor for 1 h and then stimulated with GM-CSF (5 ng/mL) for 4 h. OSM levels were measured with the ELISA method. B—PBS-treated and unstimulated cells; C—PBS-treated and GM-CSF-stimulated cells; Wort—50 μM of wortmannin-treated and GM-CSF-stimulated cells; MK 2206—50 μM of MK 2206-treated and GM-CSF-stimulated cells; PDTC—100 μM of PDTC-treated and GM-CSF-stimulated cells. Data are presented as the mean ± S.E.M. of three independent experiments. * *p* < 0.05 vs. the PBS-treated and GM-CSF-stimulated cells.

Next, we examined the mechanisms of OSM elevation. Because Su et al. [28] suggested that OSM was produced through PI3K/Akt/NF-κB signal cascades in osteoblasts, we measured the production levels of OSM using a specific inhibitor for each component of the cascade. Indeed, all inhibitors decreased the production levels of OSM in the supernatant of GM-CSF-stimulated neutrophil-like dHL-60 cells (Figure 2). PI3K inhibitor wortmannin (50 μM), Akt inhibitor MK 2206 (50 μM), and NF-κB inhibitor PDTC (100 μM) significantly reduced the production levels of OSM, indicating a dependency of PI3K/Akt/NF-κB signal cascades on OSM production. The concentrations of each inhibitor were selected considering previous reports [29,30,31].

### 2.3. GM-CSF Stimulation Results in Phosphorylation of PI3K or Akt or NF-κB in Neutrophil-like dHL-60 Cells

**Figure 3:**
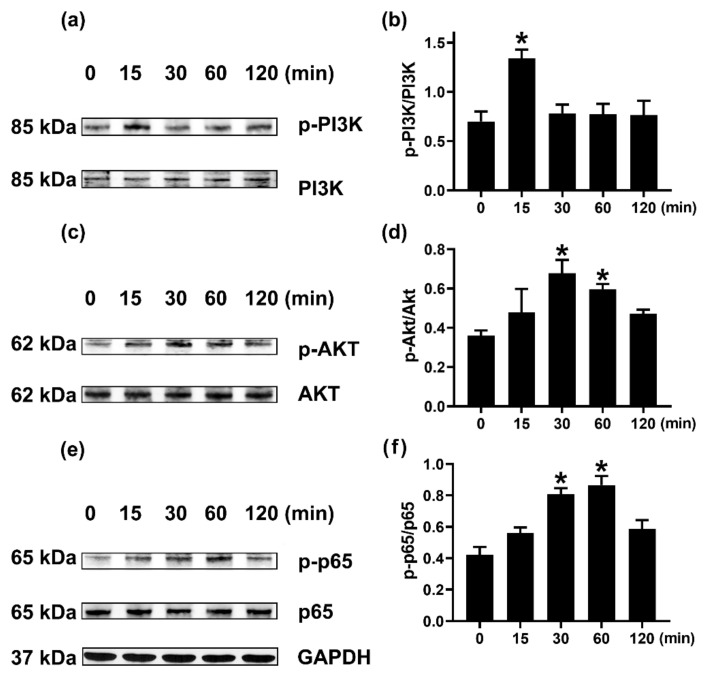
Phosphorylations of PI3K, Akt, and NF-κB in neutrophil-like dHL-60 cells. (**a**,**c**,**e**) dHL-60 cells (5 × 10^6^) were stimulated with GM-CSF (5 ng/mL). (**b**,**d**,**f**) The protein expression levels were quantitated by densitometry. Data are presented as the mean ± S.E.M. of three independent experiments. * *p* < 0.05 vs. the 0 min group.

We investigated the mechanism of OSM elevation. To confirm the dependency of PI3K/Akt/NF-κB signal cascades on OSM production, we investigated when PI3K, Akt, and NF-κB respectively reached the maximum phosphorylation after GM-CSF stimulation in neutrophil-like dHL-60 cells. PI3K reached the maximum phosphorylation 15 min after GM-CSF stimulation (Figure 3a,b). Akt reached the maximum phosphorylation 30 min after GM-CSF stimulation (Figure 3c,d). Lastly, NF-κB reached the maximum phosphorylation 60 min after GM-CSF stimulation (Figure 3e,f).

### 2.4. Dex Inhibits OSM Elevations in Neutrophil-like dHL-60 Cells

**Figure 4:**
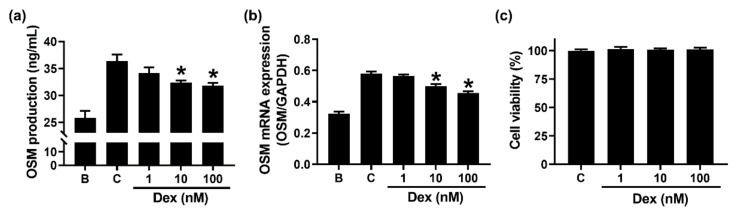
Effects of Dex on the production and mRNA expression of OSM in neutrophil-like dHL-60 cells. (**a**) dHL-60 cells (5 × 10^5^) were pretreated with Dex (1 to 100 nM) for 1 h and then stimulated with GM-CSF (5 ng/mL) for 4 h. (**b**) dHL-60 cells (1 × 10^6^) were pretreated with Dex (1–100 nM) for 1 h and then stimulated with GM-CSF (5 ng/mL) for 1 h. (**c**) Cell viability was analyzed by an MTT assay. B—PBS-treated and unstimulated cells; C—PBS-treated and GM-CSF-stimulated cells. Data are presented as the mean ± S.E.M. of three independent experiments. * *p* < 0.05 vs. the PBS-treated and GM-CSF-stimulated cells.

To examine whether Dex could inhibit the production and mRNA expression of OSM in neutrophil-like dHL-60 cells, we pretreated Dex for 1 h. The pretreatment with Dex resulted in a decrease in OSM production (Figure 4a). In addition, Dex dose-dependently downregulated the expression of OSM mRNA (Figure 4b). Cytotoxicity was not shown in Dex-treated cells (Figure 4c). Because the inhibitory effect at 100 nM of Dex was better than that at 1 and 10 nM, we examined the effect of Dex at 100 nM in the subsequent experiments (effects of Dex on the phosphorylation of PI3K or Akt or NF-κB).

### 2.5. Dex Reduces Phosphorylation of PI3K or Akt or NF-κB in Neutrophil-like dHL-60 Cells

**Figure 5:**
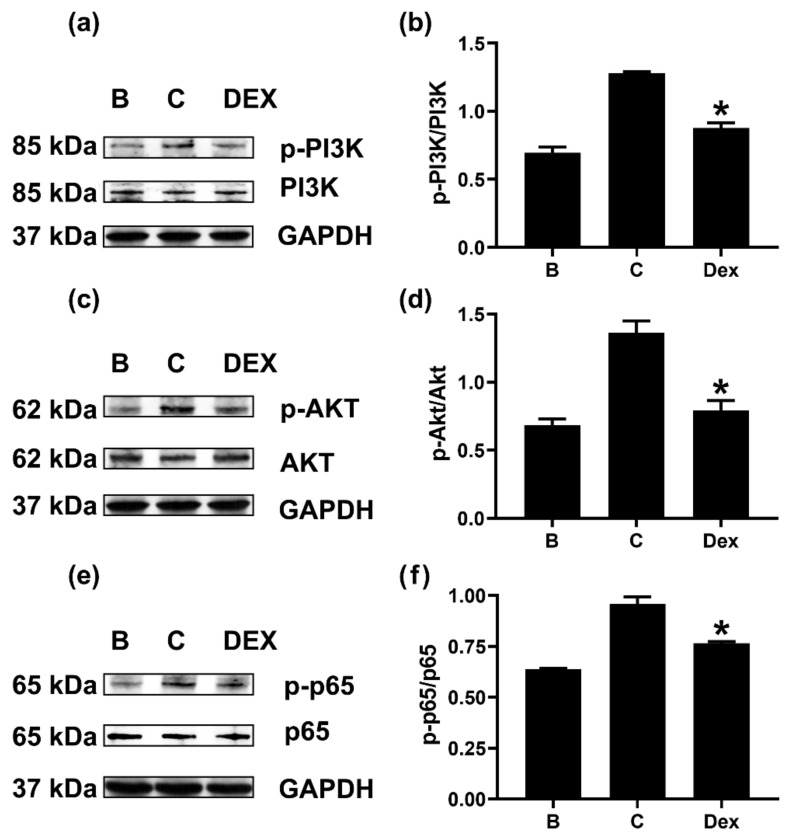
Effects of Dex on the phosphorylations of PI3K, Akt, and NF-κB in neutrophil-like dHL-60 cells. (**a**,**c**,**e**) dHL-60 cells (5 × 10^6^) were pretreated with Dex (100 nM) for 1h and then stimulated with GM-CSF (5 ng/mL) for 15 min (PI3K), 30 min (Akt), or 60 min (p65). (**b**,**d**,**f**) The protein expression levels were quantitated by densitometry. B—PBS-treated and unstimulated cells; C—PBS-treated and GM-CSF-stimulated cells; Dex—Dex-treated and GM-CSF-stimulated cells. Data are presented as the mean ± S.E.M. of three independent experiments. * *p* < 0.05 vs. the PBS-treated and GM-CSF-stimulated cells.

To evaluate the effect of Dex on the above-established signaling pathways (PI3K/Akt/NF-κB), we pretreated neutrophil-like dHL-60 cells with Dex for 1 h. The pretreatment with Dex induced downregulation of PI3K phosphorylation (Figure 5a,b). Additionally, phosphorylation of Akt was reduced by Dex treatment (Figure 5c,d). Finally, Dex pretreatment resulted in a decrease in phosphorylated levels of NF-κB in neutrophil-like dHL-60 cells (Figure 5e,f). To know whether Dex treatment can affect the mRNA levels of PI3K, Akt, and NF-κB, we performed quantitative real-time PCR. Dex treatment affected the mRNA levels of NF-κB, but not PI3K and Akt (Figure S1).

### 2.6. Dex Inhibits OSM-Induced Inflammatory Reactions in HaCaT Cells

**Figure 6:**
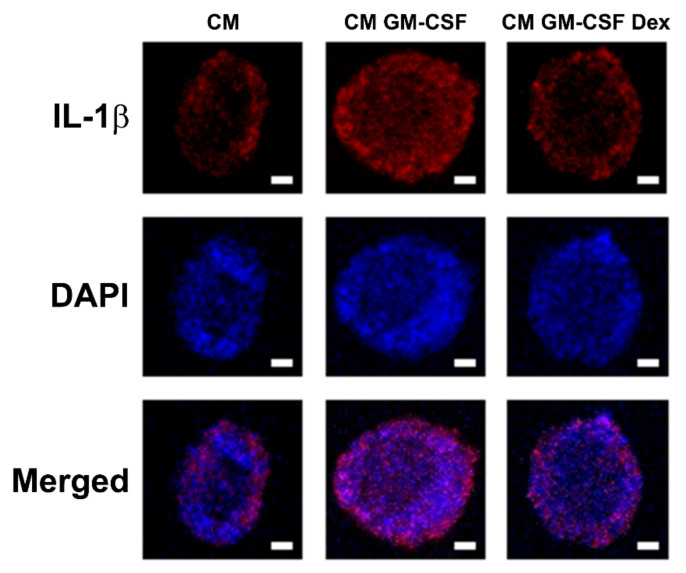
Effects of Dex on the expression of IL-1β in CM-treated HaCaT cells. HaCaT cells were incubated with each medium (CM—CM from PBS-treated and unstimulated dHL-60 cells; CM GM-CSF—CM from PBS-treated and GM-CSF-stimulated dHL-60 cells; CM GM-CSF Dex—CM from Dex-treated and GM-CSF-stimulated dHL-60 cells) for 12 h. Fluorescence microscope images stained with an anti-IL-1β antibody (scale bar = 2 μm). Representative images were obtained from images performed in triplicate.

To examine how suppression of OSM reduces inflammation, we confirmed the contribution of OSM to inflammatory reactions in HaCaT cells. Similar to the report of Liu et al. [11], treatment with OSM resulted in increases in IL-1β levels in HaCaT cells (Figure S2a). To evaluate the effect of Dex, we incubated HaCaT cells with dHL-60 cells-conditioned medium (CM) for 12 h. The incubation with CM GM-CSF induced increased IL-1β expression in HaCaT cells; however, this increase was lowered by Dex (Figure 6). In addition, IL-1β levels in the supernatants from CM GM-CSF (i.e., without Dex)-treated HaCaT cells were significantly elevated (Figure S2b). However, the elevated IL-1β levels were lowered by Dex treatment, suggesting suppression of inflammation by Dex (Figure S2b).

## 3. Discussion

High OSM levels were shown in inflammatory disorders, such as chronic rhinosinusitis and asthma [32]. Ma and colleagues [33] reported that GM-CSF treatment results in an increase in OSM mRNA expression. Furthermore, several studies suggested that OSM upregulation was induced by GM-CSF addition in isolated human neutrophils [10,26,27,34]. Our result also showed that GM-CSF addition induced OSM upregulation in neutrophil-like dHL-60 cells (Figure 1). The OSM upregulation was abolished by Dex treatment (Figure 4). Similar to our results, Grenier et al. [34] presented that Dex treatment decreased OSM production in human polymorphonuclear neutrophils stimulated by GM-CSF plus LPS. However, Grenier et al. [34] just showed the production of OSM. We provided the precise molecular mechanism of OSM production. Furthermore, Grenier et al. [34] showed the effects of Dex in LPS plus GM-CSF-stimulated neutrophils, whereas we showed the effects of Dex in only GM-CSF-stimulated cells. Substantial increases in mRNA and protein of OSM were shown in patients with asthma, while no OSM levels were detected in control subjects [35]. OSM knockout mice and OSM neutralization repressed colitis severity [2]. Thus, we presume that Dex treatment would be helpful to repress inflammatory disorders through down-regulation of OSM. In the present study, we just evaluated the pretty short periods of time (~4 h) of the effects—the OSM production under GM-CSF, GM-CSF + inhibitors, or Dex + GM-CSF treatment. To provide more information for the study, especially the anti-inflammatory effects of Dex, we assessed OSM levels at a long time point (12 h). Unfortunately, significant elevation of OSM was not induced 12 h after GM-CSF stimulation (Figure 1a).

PI3K is known to play an essential role in regulating various intracellular signaling events [36]. Akt (a downstream molecule of PI3K) is a serine/threonine kinase that has an important role in inflammatory reactions [37]. The PI3K/Akt signal cascade is crucial in triggering and amplifying the cytokine network [38]. The PI3K/AKT signaling pathway is believed to play an important role in various diseases, including cancer, cardiovascular diseases, and inflammation [39]. Akt activation resulted in NF-κB activation [38]. NF-κB is known as a downstream transcription factor of PI3K/Akt [38] and is also regarded as a critical transcription factor in chronic inflammatory responses [28]. Su and colleagues [28] suggested that OSM was produced through PI3K/Akt/NF-κB signal cascades in osteoblasts. Our results showed that each inhibitor reduced OSM production levels, suggesting a dependency of PI3K/Akt/NF-κB signal cascades on OSM production (Figure 2). To our knowledge, this is the first study showing the dependency of the PI3K signaling pathway in the production of OSM in neutrophil-like dHL-60 cells. In the present study, Dex treatment impaired phosphorylation of PI3K or Akt or NF-κB (Figure 5). Thus, it is possible that downregulation of OSM by Dex, at least in part, would be mediated by PI3K/Akt/NF-κB signal cascades in neutrophil-like dHL-60 cells. On the other hand, the inhibition of OSM production and mRNA expression was slightly weaker than the inhibition of phosphorylated levels of PI3K, Akt, and NF-κB. Thus, we can presuppose another mechanism.

OSM is secreted by T cells, macrophages, monocytes, dendritic cells, and neutrophils [2,3,4]. Secreted OSM induced inflammatory cytokine and chemokine expression in endothelial cells [13]. Treatment with recombinant OSM resulted in elevated inflammatory reactions in human intestinal stroma and skin [2,4]. OSM stimulation promoted human keratinocyte-mediated inflammation [11]. We confirmed a contribution of OSM in inflammatory reaction through upregulation of IL-1β production in OSM-stimulated HaCaT cells (Figure S2a). Incubation of HaCaT cells with CM GM-CSF increased IL-1β expression (Figure 6). This increased IL-1β expression was lowered by Dex treatment (i.e., CM GM-CSF Dex) (Figure 6). Thus, we assume that suppression of OSM may reduce inflammation.

Influenza virus H3N2 infection resulted in high levels of OSM mRNA in human nasal epithelial cells [40]. Hence, we estimate that downregulation of OSM by Dex treatment might be helpful to treat pulmonary inflammatory diseases as well as viral diseases. Our hypothesis is supported by the report of Horby et al. [41]. Daily administration of Dex (1 mg/kg = approximately 2.55 μM) for two weeks showed no toxic properties in rats [42]. A clinical dose (0.1 mg/mL = approximately 255 μM) of Dex did not exhibit any toxicity in an in vitro model [43]. In this study, we used 100 nM of Dex, and we presume that this dose would not be toxic to humans. Babatunde et al. [44] demonstrated that differentiated HL-60 neutrophil-like cells (dHL-60) have different features with respect to primary neutrophils. However, the study of Babatunde et al. [44] focused on chemotaxis and swarming of differentiated HL-60 neutrophil-like cells. HL-60 is a commonly used substitute cell line model to study neutrophil functions because of the short life span and donor variability of primary neutrophils. Hence, we used dHL-60 cells to clarify the molecular mechanism of OSM production.

**Figure 7:**
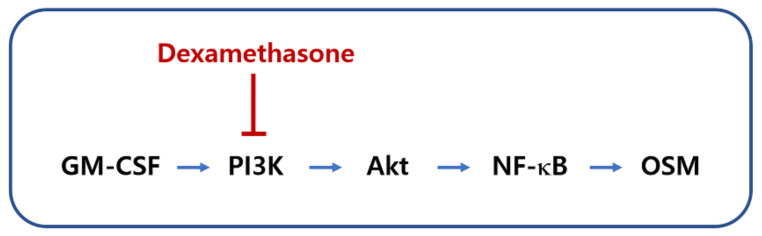
A schematic diagram of the proposed regulation of OSM by Dex.

In conclusion, we provided the underlying mechanisms of OSM production and showed that Dex suppressed the production of OSM through blocking of the PI3K/Akt/NF-κB signaling pathway in neutrophil-like dHL-60 cells (Figure 7). Our findings suggest the potential of Dex in the treatment of inflammatory diseases.

## 4. Materials and Methods

### 4.1. Materials

Dex (C_22_H_29_FO_5_, Sigma-Aldrich Co., Burlington, MA, USA) was dissolved in distilled water (DW) and further diluted with culture media. OSM antibodies, recombinant human OSM, and granulocyte-macrophage colony-stimulating factor (GM-CSF) were purchased from R&D Systems (Minneapolis, MN, USA). Phosphatidylinositol 3-kinase (PI3K) p85, phosphorylated (p)-Akt (Ser 473), Akt, p-NF-κB p65 (Ser 536), NF-κB p65, and GAPDH antibodies were purchased from Santa Cruz Biotechnology (Santa Cruz, CA, USA). p-PI3K p85 (Tyr 467) was purchased from Cell Signaling Technology (Danvers, MA, USA).

### 4.2. Cells

HL-60 cells (growth pattern: suspension) were cultured in RPMI 1640 (Gibco BRL, Grand Island, NY, USA) containing 10% (*v/v*) heat-inactivated fetal bovine serum (FBS) (Welgene, Daegu, Korea), 100 IU/mL penicillin, and 100 µg/mL streptomycin. HL-60 cells were differentiated with 1.3% DMSO for 7 days to generate the neutrophilic phenotype dHL-60. The dHL-60 cells were stimulated with 5 ng/mL of recombinant human GM-CSF by referring to a report by Elbjeirami et al. [28]. On the other hand, 4 h after GM-CSF stimulation, dHL-60 cells-conditioned medium (CM) was collected by centrifugation for immunofluorescence experiments. HaCaT cells were incubated with 2 mL of each medium (CM—CM from PBS-treated and unstimulated dHL-60 cells; CM GM-CSF—CM from PBS-treated and GM-CSF-stimulated dHL-60 cells; CM GM-CSF Dex—CM from Dex-treated and GM-CSF-stimulated dHL-60 cells) in 6-well plates for 12 h. Twelve hours after incubation, collected supernatants were used for IL-1β assay, whereas harvested HaCaT cells were used for immunofluorescence experiments (Figure S3).

### 4.3. Cell Viability Assay

The dHL-60 cells (1 × 10^5^) were pretreated with Dex or PBS for 1 h and stimulated with GM-CSF for 4 h. The cell viability was assessed as previously described [45,46,47,48,49].

### 4.4. Measurements of OSM Production

The dHL-60 cells (5 × 10^5^) were pretreated with Dex or PBS for 1 h and stimulated with GM-CSF for 4 h. OSM levels were assessed by means of an enzyme-linked immunosorbent assay, as previously described [45,50].

### 4.5. Quantitative Real-Time PCR

The dHL-60 cells (1 × 10^6^) were pretreated with Dex or PBS for 1 h and stimulated with GM-CSF for 1 h. OSM mRNA expression was assessed as previously described [51,52,53,54,55].

### 4.6. Western Blot Analysis

The dHL-60 cells (5 × 10^6^) were pretreated with Dex or PBS for 1 h and stimulated with GM-CSF for 15 min (PI3K) or 30 min (Akt) or 60 min (NF-κB). Western blot analysis was conducted as previously described [56,57,58].

### 4.7. Staining Analysis

HaCaT cells were fixed with 4% paraformaldehyde, permeabilized in 0.2% Triton X-100, and incubated with a blocking buffer to reduce nonspecific binding. The staining analysis was performed as previously described [59,60].

### 4.8. Statistical Analysis

One-way ANOVA followed by a Tukey post hoc test and independent T-test was used to assess the statistically significant differences between the means (SPSS v23 statistics software, Armonk, NY, USA). The difference is significant if the *p*-value is < 0.05.

## Data Availability

Data are contained within the article.

## Supplementary Materials

Figure S1: Effects of Dex on the mRNA expressions of PI3K, Akt, and PI3K in neutrophil-like dHL-60 cells. Figure S2: IL-1β production and effects of Dex in dHL-60 cells-conditioned medium (CM)-treated HaCaT cells. Figure S3: Experimental protocol for immunofluorescence and IL-1β experiments.

## Sample Availability

Samples of the compounds are available from the authors.
