# Correction: Han et al. Dexamethasone Attenuates Oncostatin M Production via Suppressing of PI3K/Akt/NF-κB Signaling in Neutrophil-like Differentiated HL-60 Cells. *Molecules* 2022, *27*, 129

**DOI:** 10.3390/molecules28052331

**Published:** 2023-03-02

**Authors:** Na-Ra Han, Seong-Gyu Ko, Hi-Joon Park, Phil-Dong Moon

**Affiliations:** 1 College of Korean Medicine, Kyung Hee University, Seoul 02447, Republic of Korea; 2 Korean Medicine-Based Drug Repositioning Cancer Research Center, College of Korean Medicine, Kyung Hee University, Seoul 02447, Republic of Korea; 3 Department of Preventive Medicine, College of Korean Medicine, Kyung Hee University, Seoul 02447, Republic of Korea; 4 Department of Anatomy & Information Sciences, College of Korean Medicine, Kyung Hee University, Seoul 02447, Republic of Korea; 5 Center for Converging Humanities, Kyung Hee University, Seoul 02447, Republic of Korea

In the original article [1], there was a mistake in Figure 1 as published. The authors want to change the time in Figure 1b from 15 min, 30 min, 45 min, 1 h, 2 h, 3 h, and 4 h to 5 min, 10 min, 15 min, 30 min, 1 h, 2 h, and 3 h. The corrected Figure 1 appears below. The authors apologize for any inconvenience caused and state that the scientific conclusions are unaffected. The original article has been updated.

**The Original Figure 1:**




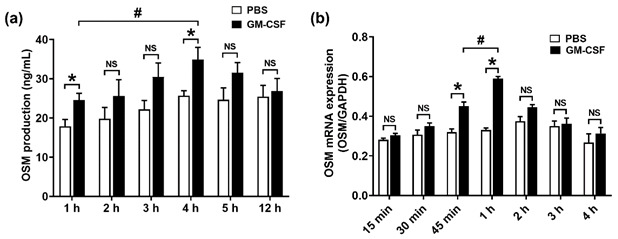




**The Correct Figure 1**:

**Figure 1:**
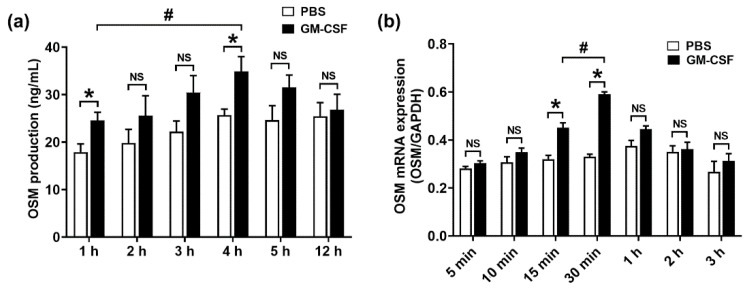
Production and mRNA expression of OSM in neutrophil-like dHL-60 cells. (**a**) dHL-60 cells (5 × 10^5^) were stimulated with GM-CSF (5 ng/mL). OSM levels were measured with the ELISA method. (**b**) dHL-60 cells (1 × 10^6^) were stimulated with GM-CSF (5 ng/mL). OSM mRNA levels were measured with the real-time PCR method. PBS—PBS-treated and unstimulated cells; GM-CSF—PBS-treated and GM-CSF-stimulated cells. Data are presented as the mean ± S.E.M. of three independent experiments. * *p* < 0.05 vs. the PBS-treated and unstimulated cells. ^#^ *p* < 0.05 vs. the indicated group. NS, not significant.
