# Supplementary material for: Dexamethasone Attenuates Oncostatin M Production via Suppressing of PI3K/Akt/NF-κB Signaling in Neutrophil-like Differentiated HL-60 Cells
Source: Molecules. 2021 Dec 27;27(1):129. doi: 10.3390/molecules27010129 (PMC8746434; doi:10.3390/molecules27010129)
Supplement: Supplementary file 1 [file molecules-27-00129-s001.zip › molecules-1531158-supplementary.pdf]

Supplementary

Dexamethasone attenuates oncostatin M production via suppressing of PI3K/Akt/NF-κB signaling in neutrophil-like differentiated HL-60 cells

Na-Ra Han [1,2], Seong-Gyu Ko [2,3], Hi-Joon Park [4] and Phil-Dong Moon [5,*]

[1] College of Korean Medicine, Kyung Hee University, 26, Kyungheedae-ro, Dongdaemun-gu, Seoul, 02447, Republic of Korea; nrhan@khu.ac.kr
[2] Korean Medicine-Based Drug Repositioning Cancer Research Center, College of Korean Medicine, Kyung Hee University, 26, Kyungheedae-ro, Dongdaemun-gu, Seoul, 02447, Republic of Korea
[3] Department of Preventive Medicine, College of Korean Medicine, Kyung Hee University, 26, Kyungheedae-ro, Dongdaemun-gu, Seoul, 02447, Republic of Korea; epiko@khu.ac.kr
[4] Department of Anatomy & Information Sciences, College of Korean Medicine, Kyung Hee University, 26, Kyungheedae-ro, Dongdaemun-gu, Seoul, 02447, Republic of Korea; acufind@khu.ac.kr
[5] Center for Converging Humanities, Kyung Hee University, 26, Kyungheedae-ro, Dongdaemun-gu, Seoul, 02447, Republic of Korea; pdmoon@khu.ac.kr
* Correspondence: pdmoon@khu.ac.kr; Tel.: +82-2-961-0897

Results

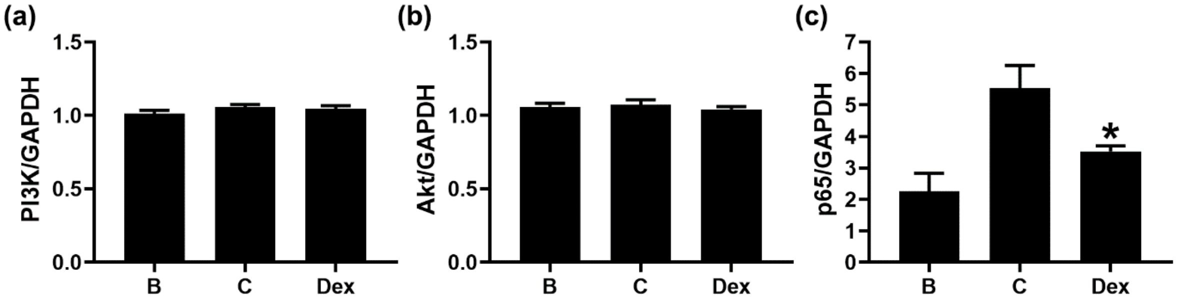

Figure S1. Effects of Dex on the mRNA expressions of PI3K, Akt, and PI3K in neutrophil-like dHL-60 cells. (a-c) dHL-60 cells (1×10^6) were pretreated with Dex (100 nM) for 1 h, and then stimulated with GM-CSF (5 ng/mL) for 15min (PI3K), 30 min (Akt), and 60 min (NF-κB). B, PBS-treated, and unstimulated cells; C, PBS-treated, and GM-CSF-stimulated cells; Dex, Dex-treated, and GM-CSF-stimulated cells. Data are presented as the mean ± S.E.M. of three independent experiments. $^*p < 0.05$ vs. the PBS-treated, and GM-CSF-stimulated cells.

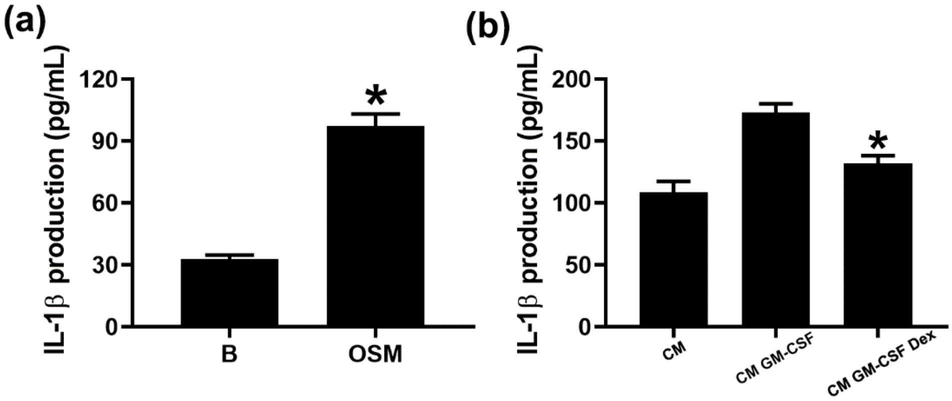

Figure S2. IL-1β production and effects of Dex in dHL-60 cells-conditioned medium (CM)-treated HaCaT cells. (**a**) HaCaT cells were stimulated with recombinant human OSM (10 ng/mL) for 12 h by referring to a report by Liu et al. [1]. B, unstimulated cells; C, OSM-stimulated cells. (b) HaCaT cells were incubated with each CM for 12 h. CM, CM from PBS-treated, and unstimulated dHL-60 cells; CM GM-CSF, CM from PBS-treated, and GM-CSF-stimulated dHL-60 cells; CM GM-CSF Dex, CM from Dex-treated, and GM-CSF-stimulated dHL-60 cells. IL-1β levels were measured with the ELISA method. Data are presented as the mean ± S.E.M. of three independent experiments. *$p < 0.05$ vs. the unstimulated cells or CM from PBS-treated, and GM-CSF-stimulated dHL-60 cells.

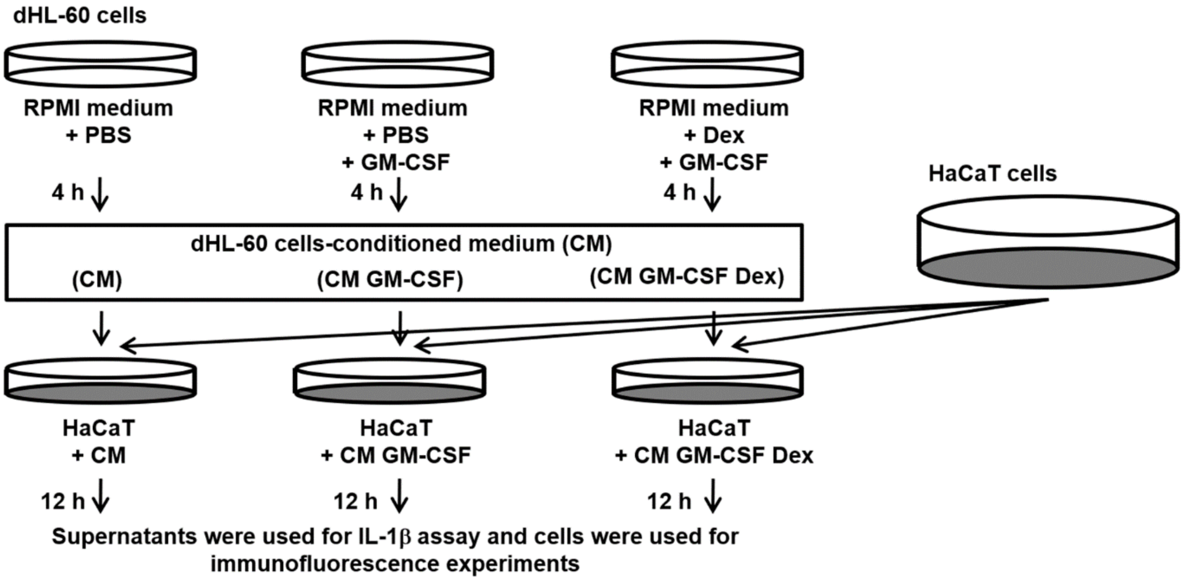

Figure S3. Experimental protocol for immunofluorescence and IL-1β experiments. CM, CM from PBS-treated, and unstimulated dHL-60 cells; CM GM-CSF, CM from PBS-treated, and GM-CSF-stimulated dHL-60 cells; CM GM-CSF Dex, CM from Dex-treated, and GM-CSF-stimulated dHL-60 cells.

Materials and Methods

Cell Viability Assay

The dHL-60 cells (1×10^5) were seeded in 24-well plate and pretreated with Dex or PBS for 1 h, and then stimulated with GM-CSF for 4 h. The cells were incubated with 3-(4,5-dimethylthiazol-2-yl)-2,5-diphenyltetrazolium bromide (MTT, Sigma-Aldrich Co.) solution at 37 °C for 4 h. Next, we added 1 mL of dimethyl sulfoxide to dissolve the MTT formazan, and transferred 100 μL of supernatant into a new

96-well microplate. A microplate reader (540 nm, Versa Max, Molecular Devices, Sunnyvale, CA, USA) was used to measure the absorbance of formazan dissolved in DMSO [2-6].

Measurements of Cytokines

Cytokines levels were assessed by means of an enzyme-linked immunosorbent assay, as previously described [2,7]. The capture antibody (R&D system Inc., Minneapolis, MN, USA) was pre-coated in a 96-well plate. Phosphate-buffered saline (PBS) containing 10% FBS was added to block the plate for 2 h. After washing the plate by means of PBS containing Tween 20 (PBST), cell supernatants were added into the plate for 2 h. After washing the plate with PBST, the plate was treated with biotinylated detection antibody (R&D system Inc.) for 2 h and then incubated with avidin-conjugated to horseradish peroxidase (Sigma-Aldrich Co.) for 30 min. Absorbance by TMB substrate (BD Pharmingen, San Jose, CA, USA) was measured by a microplate reader (405 nm, Versa Max).

Quantitative real-time PCR

The dHL-60 cells (1×10^6) were seeded in 6-well plate and pretreated with Dex or PBS for 1 h, and then stimulated with GM-CSF for 1 h. The harvested cells were used to isolate total RNA by means of an RNA extraction reagent (iNtRON, Seongnam, Republic of Korea), as previously described [8-12]. The first-strand cDNA from total RNA was synthesized with cDNA synthesis reagents (Bioneer, Daejeon, Republic of Korea). The following designed primers were used for the real time PCR (Applied Biosystems, Foster City, CA, USA) by using Power SYBR® Green Master Mix (Applied Biosystems): *OSM*: 5′- GCTCACACAGAGGACGCTG-3′, 5′- GGAGCACGCGGTACTCTTTC-3′; *PI3K*: 5′-GAATCCAATGGGAACTGT-3′, 5′- GGGAGGGTAATAATAAGGT-3′; *Akt*: 5′-TGCAGCATCGCTTCTTTG-3′, 5′- TCTGGGCCGTGAACTCCT-3′; *NF-κB*: 5′-GACAGTGACAGTGTCTGCGA-3′, 5′- AGTTAGCAGTGAGGCACCAC-3′; *GAPDH*: 5′-TCGACAGTCAGCCGCATCTTCTTT-3′, 5′-ACCAAATCCGTTGACTCCGACCTT-3′. The relative expression of mRNA for OSM was normalized by GAPDH and measured by using $2^{-\Delta\Delta Ct}$ method.

Western Blot Analysis

The dHL-60 cells (5×10^6) were seeded in 60 mm dish and pretreated with Dex or PBS for 1 h, and then stimulated with GM-CSF for 15 min (PI3K) or 30 min (Akt) or 60 min (NF-κB). Western blot analysis was conducted, as previously described [13-15]. An ice-cold cell lysis buffer (Sigma-Aldrich Co.) was used to lyse the harvested cells. Cell extracts were prepared with sampling buffer (Laemmli's 2×, ELPISBIOTECH. INC., Daejeon, Republic of Korea) and heated at 95 °C for 5 min. Proteins were subjected to electrophoresis using 10% - 15% gel containing sodium dodecyl sulfate and transferred to nitrocellulose membranes (Amersham™, Chicago IL, USA). PBST containing 5% bovine serum albumin (Sigma-Aldrich Co.) was used to block the membranes afterwards relevant primary antibodies (phosphorylated (p)-PI3K, Cell Signaling Technology, Danvers, MA, USA; PI3K, p-Akt, Akt, p-p65, p65, and GAPDH, Santa Cruz Biotechnology, Santa Cruz, CA, USA) were used. Peroxidase-conjugated secondary antibodies (Santa Cruz Biotechnology) were added for incubation of the membranes for 1 h at room temperature after washing with PBST. Specific bands were detected by an enhanced chemiluminescence solution (DoGenBio Co., Seoul, Republic of Korea). Band intensities were calculated with ImageJ program (National health institute, Bethesda, MD, USA).

Staining Analysis

HaCaT cells were fixed with 4% paraformaldehyde, permeabilized in 0.2% Triton X-100, and incubated with a blocking buffer to reduce nonspecific binding. The staining analysis was performed, as previously described [16,17]. The cells were incubated with the primary antibody (anti-IL-1β), followed by incubation with Alexa Fluor® conjugated secondary antibody (Alexa Fluor® 647, Abcam, Cambridge, MA, USA) at room temperature. For nuclear staining, 4′ ,6-diamidino-2-phenylindole (DAPI) was used. Samples were visualized under a confocal laser-scanning microscope (Carl Zeiss, Oberkochen, Germany).

References

1. Liu, J.; Zhong, Y.; Liu, H.; Yang, H.; Lu, P.; Shi, Y.; Wang, X.; Zheng, W.; Yu, X.; Xu, Y.; et al. Oncostatin M sensitizes keratinocytes to UVB-induced inflammation via GSDME-mediated pyroptosis. *J. Dermatol. Sci.* **2021**, *104*, 95-103. https://doi.org/10.1016/j.jdermsci.2021.09.004

2. Moon, P.D.; Lee, J.S.; Kim, H.Y.; Han, N.R.; Kang, I.; Kim, H.M.; Jeong, H.J. Heat-treated Lactobacillus plantarum increases the immune responses through activation of natural killer cells and macrophages on in vivo and in vitro models. *J. Med. Microbiol.* **2019**, *68*, 467-474. https://doi.org/10.1099/jmm.0.000938

3. Etsassala, N.; Ndjoubi, K.O.; Mbira, T.J.; Pearce, B.; Pearce, K.; Iwuoha, E.I.; Hussein, A.A.; Benjeddou, M. Glucose-Uptake Activity and Cytotoxicity of Diterpenes and Triterpenes Isolated from Lamiaceae Plant Species. *Molecules* **2020**, *25*, 4129. https://doi.org/10.3390/molecules25184129

4. Banach, M.; Wiloch, M.; Zawada, K.; Cyplik, W.; Kujawski, W. Evaluation of Antioxidant and Anti-Inflammatory Activity of Anthocyanin-Rich Water-Soluble Aronia Dry Extracts. *Molecules* **2020**, *25*, 4055. https://doi.org/10.3390/molecules25184055

5. Wu, T.; Fang, X.; Xu, J.; Jiang, Y.; Cao, F.; Zhao, L. Synergistic Effects of Ginkgolide B and Protocatechuic Acid on the Treatment of Parkinson's Disease. *Molecules* **2020**, *25*, 3976. https://doi.org/10.3390/molecules25173976

6. Tumosienė, I.; Kantminienė, K.; Klevinskas, A.; Petrikaitė, V.; Jonuškienė, I.; Mickevičius, V. Antioxidant and Anticancer Activity of Novel Derivatives of 3-[(4-Methoxyphenyl)amino]propane-hydrazide. *Molecules* **2020**, *25*, 2980. https://doi.org/10.3390/molecules25132980

7. Moon, P.D.; Han, N.R.; Kim, H.M.; Jeong, H.J. High-Fat Diet Exacerbates Dermatitis through Up-Regulation of TSLP. *J. Invest. Dermatol.* **2019**, *139*, 1198-1201. https://doi.org/10.1016/j.jid.2018.11.003

8. Han, N.R.; Kim, H.Y.; Kang, S.; Kim, M.H.; Yoon, K.W.; Moon, P.D.; Kim, H.M.; Jeong, H.J. Chrysophanol, an anthraquinone from AST2017-01, possesses the anti-proliferative effect through increasing p53 protein levels in human mast cells. *Inflamm. Res.* **2019**, *68*, 569-579. https://doi.org/10.1007/s00011-019-01239-7

9. Han, N.R.; Moon, P.D.; Kim, H.M.; Jeong, H.J. TSLP Exacerbates Septic Inflammation via Murine Double Minute 2 (MDM2) Signaling Pathway. *J. Clin. Med.* **2019**, *8*, 1350. https://doi.org/10.3390/jcm8091350
10. Fathy, M.; Okabe, M.; M Othman, E.; Saad Eldien, H.M.; Yoshida, T. Preconditioning of Adipose-Derived Mesenchymal Stem-Like Cells with Eugenol Potentiates Their Migration and Proliferation In Vitro and Therapeutic Abilities in Rat Hepatic Fibrosis. *Molecules* **2020**, *25*, 2020. https://doi.org/10.3390/molecules25092020
11. He, L.Y.; Hu, M.B.; Li, R.L.; Zhao, R.; Fan, L.H.; Wang, L.; Peng, W.; Liu, Y.J.; Wu, C. J. The Effect of Protein-Rich Extract from Bombyx Batryticatus against Glutamate-Damaged PC12 Cells Via Regulating γ-Aminobutyric Acid Signaling Pathway. *Molecules* **2020**, *25*, 553. https://doi.org/10.3390/molecules25030553
12. Brizzolara, A.; Garbati, P.; Vella, S.; Calderoni, M.; Quattrone, A.; Tonini, G.P.; Capasso, M.; Longo, L.; Barbieri, R.; Florio, T.; et al. Co-Administration of Fendiline Hydrochloride Enhances Chemotherapeutic Efficacy of Cisplatin in Neuroblastoma Treatment. *Molecules* **2020**, *25*, 5234. https://doi.org/10.3390/molecules25225234
13. Moon, P.D.; Han, N.R.; Lee, J.S.; Kim, H.M.; Jeong, H.J. Ursolic acid downregulates thymic stromal lymphopoietin through the blockade of intracellular calcium/caspase-1/NF-κB signaling cascade in HMC-1 cells. *Int. J. Mol. Med.* **2019**, *43*, 2252-2258. https://doi.org/10.3892/ijmm.2019.4144
14. Moon, P.D.; Han, N.R.; Lee, J.S.; Hong, S.; Yoo, M.S.; Kim, H.J.; Kim, J.H.; Kang, S.; Jee, H.W.; Kim, H.M.; et al. Use of Physcion to Improve Atopic Dermatitis-Like Skin Lesions through Blocking of Thymic Stromal Lymphopoietin. *Molecules* **2019**, *24*, 1484. https://doi.org/10.3390/molecules24081484
15. Han, N.R.; Han, S.J.; Moon, P.D.; Hong, S.; Kim, H.; Li, Y.H.; Kim, H.M.; Jeong, H.J. Effect of dexamethasone injection into Zusanli (ST 36) acupoint on ovalbumin-induced allergic rhinitis. *J. Tradit. Chin. Med.* **2019**, *39*, 307-314.
16. Moon, P.D.; Kim, M.H.; Lim, H.S.; Oh, H.A.; Nam, S.Y.; Han, N.R.; Kim, M.J.; Jeong, H.J.; Kim, H.M. Taurine, a major amino acid of oyster, enhances linear bone growth in a mouse model of protein malnutrition. *BioFactors* **2015**, *41*, 190-197. https://doi.org/10.1002/biof.1213

17. Han, N.R.; Kim, K.C.; Kim, J.S.; Ko, S.G.; Park, H.J.; Moon, P.D. The im-mune-enhancing effects of a mixture of Astragalus membranaceus (Fisch.) Bunge, Angel-ica gigas Nakai, and Trichosanthes Kirilowii (Maxim.) or its active constituent nodakenin. *J. Ethnopharmacol*. **2021**, *285*, 114893. Advance online publication. https://doi.org/10.1016/j.jep.2021.114893
